# Identification of Key Genes Related to Lung Squamous Cell Carcinoma Using Bioinformatics Analysis

**DOI:** 10.3390/ijms21082994

**Published:** 2020-04-23

**Authors:** Miaomiao Gao, Weikaixin Kong, Zhuo Huang, Zhengwei Xie

**Affiliations:** 1 Peking University International Cancer Institute and Department of Pharmacology, School of Basic Medical Sciences, Peking University, Beijing 100191, China; 2 Department of Molecular and Cellular Pharmacology, School of Pharmaceutical Sciences, Peking University, Beijing 100191, China

**Keywords:** lung squamous carcinoma, bioinformatics, prognosis

## Abstract

Lung squamous cell carcinoma (LUSC) is often diagnosed at the advanced stage with poor prognosis. The mechanisms of its pathogenesis and prognosis require urgent elucidation. This study was performed to screen potential biomarkers related to the occurrence, development and prognosis of LUSC to reveal unknown physiological and pathological processes. Using bioinformatics analysis, the lung squamous cell carcinoma microarray datasets from the Gene Expression Omnibus (GEO) and The Cancer Genome Atlas (TCGA) databases were analyzed to identify differentially expressed genes (DEGs). Furthermore, PPI and WGCNA network analysis were integrated to identify the key genes closely related to the process of LUSC development. In addition, survival analysis was performed to achieve a prognostic model that accomplished good prediction accuracy. Three hundred and thirty–seven up–regulated and 119 down-regulated genes were identified, in which four genes have been found to play vital roles in LUSC development, namely CCNA2, AURKA, AURKB, and FEN1. The prognostic model contained 5 genes, which were all detrimental to prognosis. The AUC of the established prognostic model for predicting the survival of patients at 1, 3, and 5 years was 0.692, 0.722, and 0.651 in the test data, respectively. In conclusion, this study identified several biomarkers of significant interest for additional investigation of the therapies and methods of prognosis of lung squamous cell carcinoma.

## 1. Introduction

Lung cancer is among the deadliest malignancies globally and can be categorized into two main types: Small cell lung cancer (SCLC) and non–small cell lung cancer (NSCLC). NSCLC, accounting for approximately 85% of lung cancer cases, is principally divided into lung adenocarcinoma (LUAD) and lung squamous cell carcinoma (LUSC) depending on pathogenesis and histological morphology [1,2,3]. Many therapeutic methods are currently being used to treat lung cancer, such as surgical resection, chemotherapy, radiotherapy, targeted therapy, and immunotherapy. Early stage NSCLC patients often undergo surgical resection, for advanced stage patients, whose tumors cannot be surgically removed, targeted therapy or immunotherapy combined with chemotherapy has been chosen as the best way for treatment [4,5]. In recent years, targeted therapies have undergone considerable development, and some effective molecular targets have been identified, such as epidermal growth factor receptor (EGFR) and anaplastic lymphoma kinase (ALK). It has been confirmed that these targets are successful in lung adenocarcinoma, but not in lung squamous cell carcinoma, because these two major subtypes have different mutation profiles [6,7,8]. Immune check point inhibitors (Nivolumab, Pembrolizumab) in combination with carboplatin and paclitaxel is the first–line therapy for LUSC in clinical nowadays [9,10]. This strategy can significantly improve response rate and prolong progression free survival [11]. However, the treatment for immunotherapy is extremely expensive and this therapy was reported can lead to serious side effect in patients recently [12]. Therefore, in order to better diagnose and treat patients with LUSC, an investigation of novel biomarkers is required. LUSC, which accounts for 30% of cases of NSCLC, is more common in middle–aged and older men and has a high rate of metastasis and recurrence [13]. NSCLC patients are mostly diagnosed during the advanced stage, their 5-year survival rate lower than that of early stage patients [4,14,15]. Thus, further study of prognostic markers is required so as to personalize cancer treatment. Although a number of studies have reported on LUSC-related genes and prognostic markers, the specific molecular mechanisms in the pathogenesis and progression of LUSC have not been systematically evaluated, which constrains the potential for early diagnosis and treatment [16,17]. An in-depth understanding of the molecular mechanisms involved in the occurrence and development of lung cancer may provide a more effective strategy for early detection and subsequent clinical treatment. Therefore, novel promising biomarkers or potential drug treatments are urgently needed.

**Figure 1:**
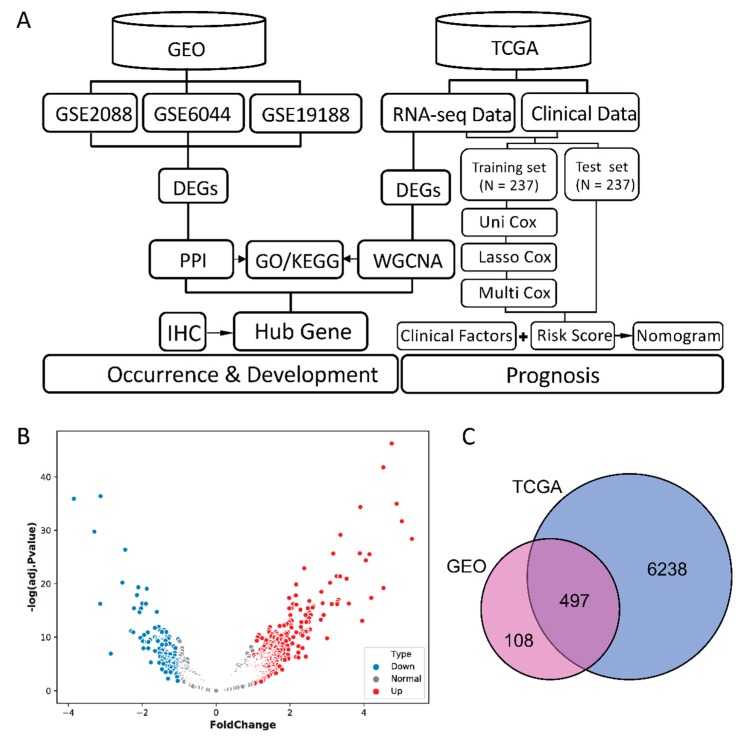
(**A**) Flow chart of the present study. (**B**) Volcano plots of differentially expressed genes (DEGs) in Gene Expression Omnibus (GEO) samples. X–axis represents the fold change of gene expression, and y-axis stands for adjusted *p* value. The red and blue dots in the plot represent statistically significant up- and down-regulated genes. (**C**) Venn diagrams of DEGs of the GEO datasets and The Cancer Genome Atlas (TCGA) dataset.

Genomic microarrays and high-throughput sequencing technology combined with bioinformatics analysis has gradually become a powerful tool for the discovery of disease biomarkers and related pathways. In the present study, three RNA microarray datasets from the Gene Expression Omnibus (GEO) database and RNAseq data from the The Cancer Genome Atlas (TCGA) database were analyzed to identify differentially expressed genes (DEGs) in lung squamous cell carcinoma compared with normal tissues. Then, protein–protein interaction (PPI) network analysis was performed to get key genes using these DEGs. On the other hand, DEGs of TCGA database were also used to weighted gene co–expression network analysis (WGCNA) so as to obtain key genes associated with the occurrence and development of LUSC. Gene ontology (GO) and Kyoto Encyclopedia of Genes (KEGG) analyses were performed to identify biological functions and pathways which possibly influence the pathogenesis of LUSC. Eventually, key genes at the intersection of these two networks analysis identified hub genes that are critical to the disease. In addition, we utilized patient clinical information from DEGs in the TCGA for univariate cox regression analysis, lasso regression analysis and multivariate cox regression analysis to identify prognostic-related key genes. This ensured that an improved understanding of the mechanism and prognosis of lung squamous cell carcinoma was obtained. The overview of this study was shown in Figure 1A.

## 2. Results

### 2.1. Identification of DEGs

Hierarchical clustering was firstly employed to detect sample groups and remove data deviating from the sample group. After measuring the quality of samples in each group, in total there were 97 normal lung samples and 84 with LUSC (Supplementary Table S1). Batch correction was performed to eliminate the batch effect of three datasets GSE2088, GSE6044, and GSE19188 (the hierarchical clustering of all samples is shown in Supplementary Figure S1). Then, 486 significantly up–regulated DEGs and 119 significantly down–regulated DEGs in merged GEO microarray datasets were identified (Figure 1B shows the volcano plot of GEO samples). In TCGA dataset, containing 49 normal samples and 499 LUSC samples, 3348 up-regulated genes and 3387 down-regulated genes were identified. The intersection is shown in Figure 1C, including 337 significantly up-regulated and 119 down–regulated genes, the change in direction of expression of TCGA consistent with the DEGs in the GEO datasets. These genes were used to perform subsequent PPI analysis.

### 2.2. PPI Network Analysis of DEGs

**Figure 2:**
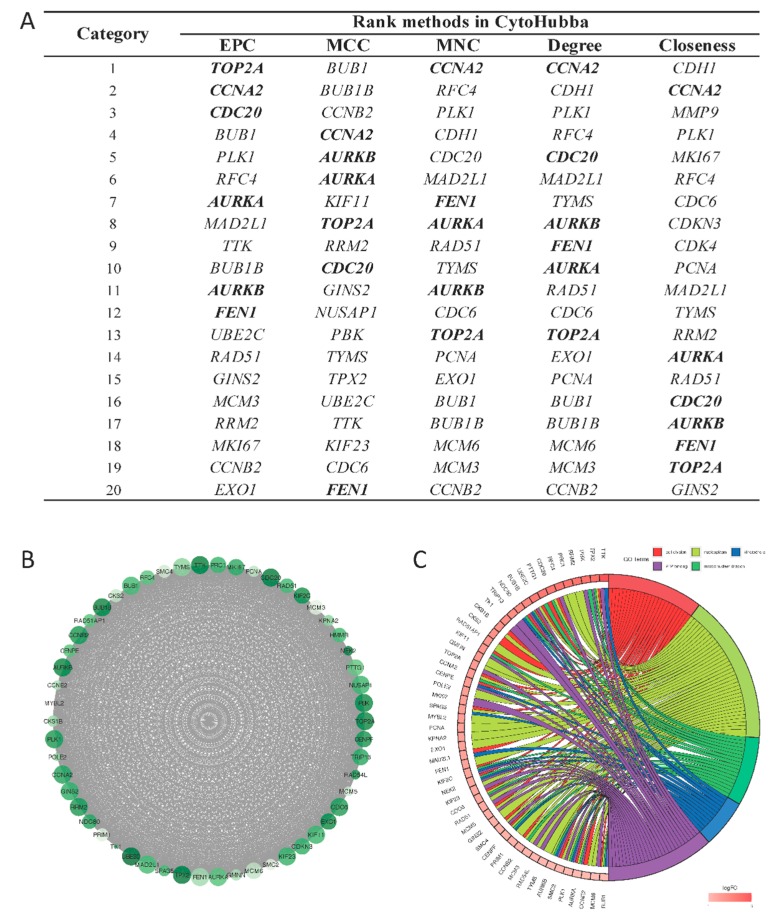
(**A**) Hub genes for highly expressed genes ranked by different CytoHubba methods. Bold gene symbols were the overlap genes in top 20 by five ranked methods. EPC: Edge percolated component; MCC: Maximal cilque centrality; MNC: Maximal neighborhood component; Degree: Node connect degree, Closeness: Node connect closeness. (**B**) The most significant module of the protein–protein interaction (PPI) network. Node size is positively related to degree of expression and the gradation of color positively associated with the expression level of this gene. (**C**) Gene ontology (GO) analysis of the most significant module in PPI analysis.

The PPI network was constructed by Cytoscape based on the STRING database, consisting of 476 nodes and 4347 edges, including 362 up- and 114 down-regulated genes (Supplementary Figure S2A). The genes that scored in the top 20 by all five methods in CytoHubba were selected as key genes of LUSC in PPI analysis. These genes were: TOP2A, CCNA2, CDC20, AURKA, AURKB, and FEN1, which may play an important role in LUSC progression (Figure 2A). MCODE in Cytoscape was used to perform module analysis. We found that most of the top 20 genes in five methods were in module 1, which is the fairly significant module (MCODE score = 52.057) in all modules (Supplementary Table S2). This module included 54 nodes and 1380 edges (Figure 2B). Remarkably, genes in this module were all up–regulated. Functional and pathway enrichment analysis of the DEGs in this module were also conducted using DAVID. GO term enrichment analysis demonstrated that genes in this module were principally enriched in cell division and mitotic nuclear division in biological processes. Cell component analysis indicated that genes were significantly enriched in nucleoplasm, spindle and kinetochore. Molecular functional analysis demonstrated that the genes were principally involved in the binding of ATP and protein (Figure 2C). KEGG analysis suggested that the genes were mainly involved in cell cycle (Supplementary Figure S2B).

### 2.3. Weighted Gene Correlation Network Analysis of DEGs

**Figure 3:**
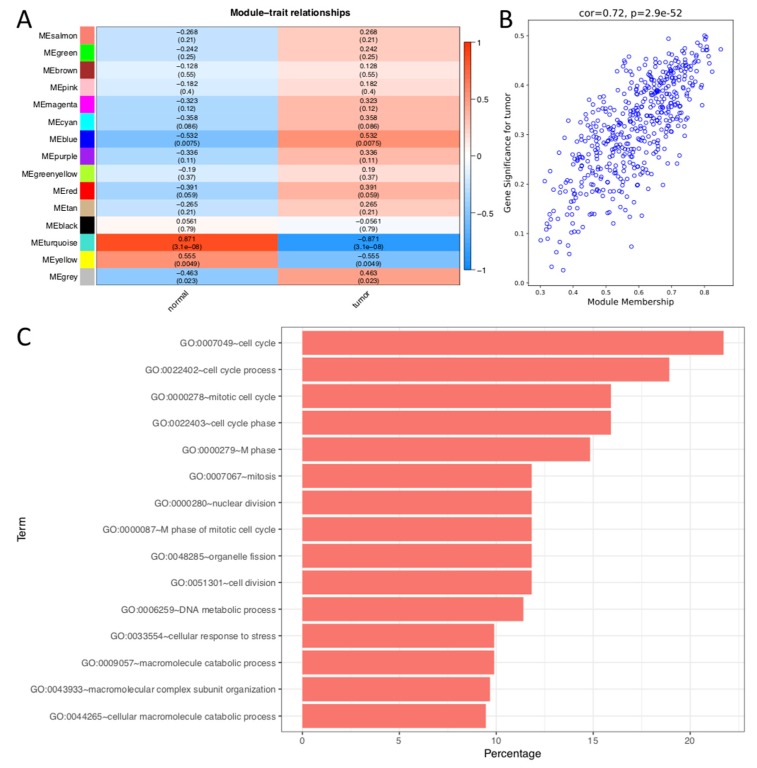
(**A**) Gene modules and phenotypes quantified using Boolean variables (1 represents occurrence, 0 represents no occurrence) were used to calculate correlation coefficients, represented as a heat map. *p*-values are displayed in brackets. (**B**) Gene correlation scatter plots of the blue module. X-axis represents molecule membership, i.e., Pearson correlation coefficients of gene and module (MM). Y-axis represents the importance of the gene for the phenotype, i.e., Pearson’s correlation coefficient of gene and phenotype (GS: phenotype is represented by a Boolean variable). In the blue module, the upper quartile value of MM and GS were 0.683 and 0.390. (**C**) GO analysis of blue module. The y-axis shows significantly enriched GO terms, and x-axis represents the percentage of gene enrichment in this term.

Based on the results of hierarchical clustering, we first removed two samples: TCGA.63.5128.01 and TCGA.92.8065.01, whose height in the hierarchical clustering tree are greater than 50,000 (Supplementary Figure S3). A value of β = 5 was selected as a soft threshold to establish a gene regulatory network (Supplementary Figure S4A). After obtaining the gene modules using a dynamic pruning method, it was found that the correlation coefficients of the blue, yellow and turquoise modules were greatest, at 0.538, -0.542, and -0.870, respectively (Figure 3A). In addition, the first principal component of the genes in these modules and the Pearson correlation coefficient between the modules in terms of clustering, were calculated. From these results (Supplementary Figure S4B), we can see that the turquoise, yellow modules were of greatest consistency. The correlation coefficients of these two modules and phenotypes were negative. MM and GS value in the upper quartile of all genes in the module were thought as the key genes of this module (The MM and GS cut-off of these three modules were shown in Supplementary Table S3. The genes distribution of blue module was shown in Figure 3B while yellow and turquoise module were shown in Supplementary Figure S4C). Interestingly, we found that of the five methods, most of top 20 genes in each method were located in the blue module. The GO (Figure 3C) and KEGG (Supplementary Figure S4D) analysis results show that the blue module was more closely related to cell cycle, mitosis, nuclear division, p53 signaling pathway, etc., which possibly related to the excessive proliferation of cells during cancer, targeted by many classic anticancer drugs such as paclitaxel and navelbine [18,19,20], that play important roles in these processes. Thus, genes in this module are important for drug development.

### 2.4. Hub Genes Related to LUSC

**Figure 4:**
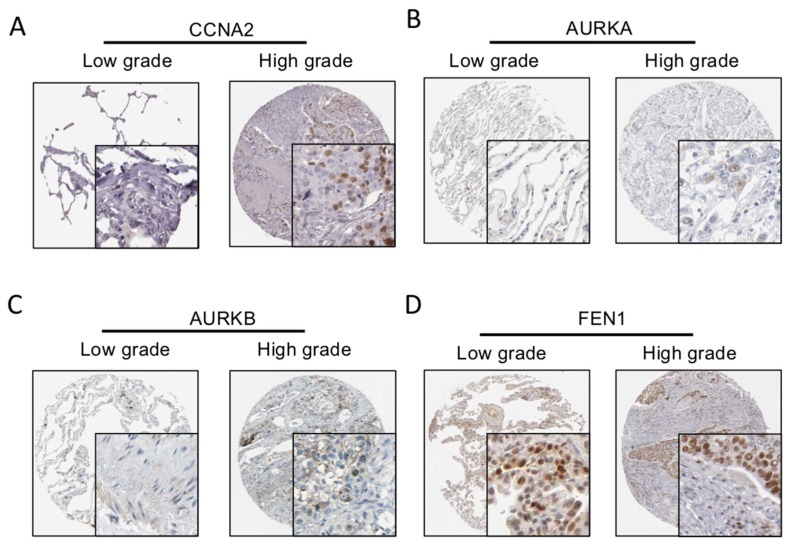
(**A**–**D**) Immunohistochemistry (IHC) validation about significant hub genes. The expression level of the four hub genes was positively correlated with disease status as they were upregulated in Lung squamous cell carcinoma (LUSC) samples. More detailed information is in Supplementary Table S5.

Key genes identified in the PPI network were mostly contained in the blue module in WGCNA analysis, including CCNA2, AURKA, AURKB, and FEN1 (Scores of these hub genes in PPI and WGCNA analysis were shown in Supplementary Table S4). GO analysis indicated that the blue module was associated with cell cycle, also consistent with PPI submodule analysis. This suggest that these four genes may play a pivotal role in LUSC development. Therefore, we defined these four genes CCNA2, AURKA, AURKB, and FEN1 as the hub genes related to LUSC. To further validation, as shown in Figure 4 (information about these samples were shown in Supplementary Table S5), immunohistochemistry (IHC) results indicated that they all have a significant up–regulated expression in LUSC (The distribution of IHC results of LUSC in whole database was shown in Supplementary Figure S5). We also analyzed whether somatic copy numbers alteration (SCNA) in LUSC are associated with high expression of these hub genes (SCNA data of LUSC were obtained in TCGA database). There is no significant correlation between hub gene’s expression and its SCNA (Supplementary Table S6).

### 2.5. Survival Analysis

**Figure 5:**
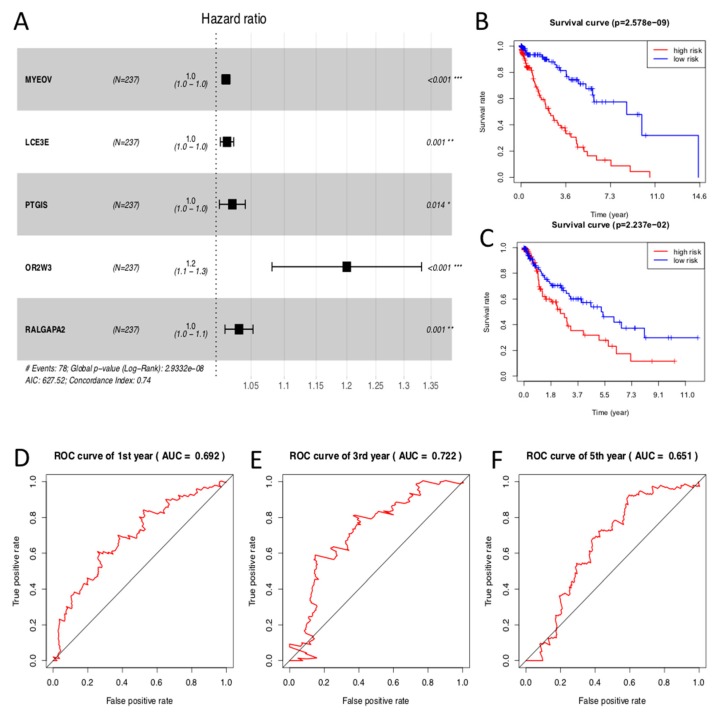
(**A**) Forest plot for multivariate cox regression. The Hazard Radio (HR) value is e^β^. This figure displays the 95% confidence interval for the HR value over the box plot with associated *p*-values. An HR of greater than 1 indicates that high gene expression is bad for the prognosis. (**B**) Survival curve for patients with different risk scores in the training data. Patients were divided into two groups according to the median survival curve score. Blue represents patients with a lower risk score. *p*-value < 0.001. (**C**) Survival curve for patients in the test data. *p*-Value < 0.05. (**D–F**) ROC curves for the model representing 1-, 3-, and 5-year predictions in test data, respectively. The values in brackets are the areas under the curve.

In order to establish an effective model for predicting prognostic status, univariate Cox proportional hazards regression analysis was employed, together with Lasso regression analysis and multivariate Cox proportional hazards regression analysis to screen genes. In the univariate Cox proportional hazards regression analysis, 91 genes with significant effects on prognosis were identified. In lasso regression, following 10-fold cross-validation with 1000 repeats, λ was 0.093 (Supplementary Figure S6A), where Partial Likelihood Deviance was the smallest. For a λ of this value, 8 of the 91 genes had coefficients that were not zero (Supplementary Figure S6B). A total of 5 genes were obtained in multivariate Cox proportional hazards regression analysis to establish a prognostic risk score model, namely MYEOV, LCE3E, PTGIS, OR2W3, RALGAPA2 (Figure 5A).

Risk score = (0.0137 × MYEOV) + (0.0152 × LCE3E) + (0.0223 × PTGIS) + (0.180 × OR2W3) + (0.0319 × RALGAPA2)(1)

The Kaplan-Meier curves were grouped by defined risk scores. We found that prognosis of the low-risk group was significantly better than that of the high-risk group in the training data (Figure 5B) and test data (Figure 5C). By predicting survival of patients at 1, 3, and 5 years, the areas under the curve (AUC) of the ROC curves obtained from the risk–based prediction model in the training data were 0.811, 0.924, and 0.937 (Supplementary Figure S7A–C) and in the test data were 0.692, 0.722, and 0.651 (Figure 5D–F). At this step, we also tested other machine learning algorithms, including Decision Tree (DT), Naïve Bayes (NB), and Random Forest (RF), to compare with the performance of our multivariate Cox model. This regression model works best among all these methods (Detailed information is in Supplementary Method).

**Figure 6:**
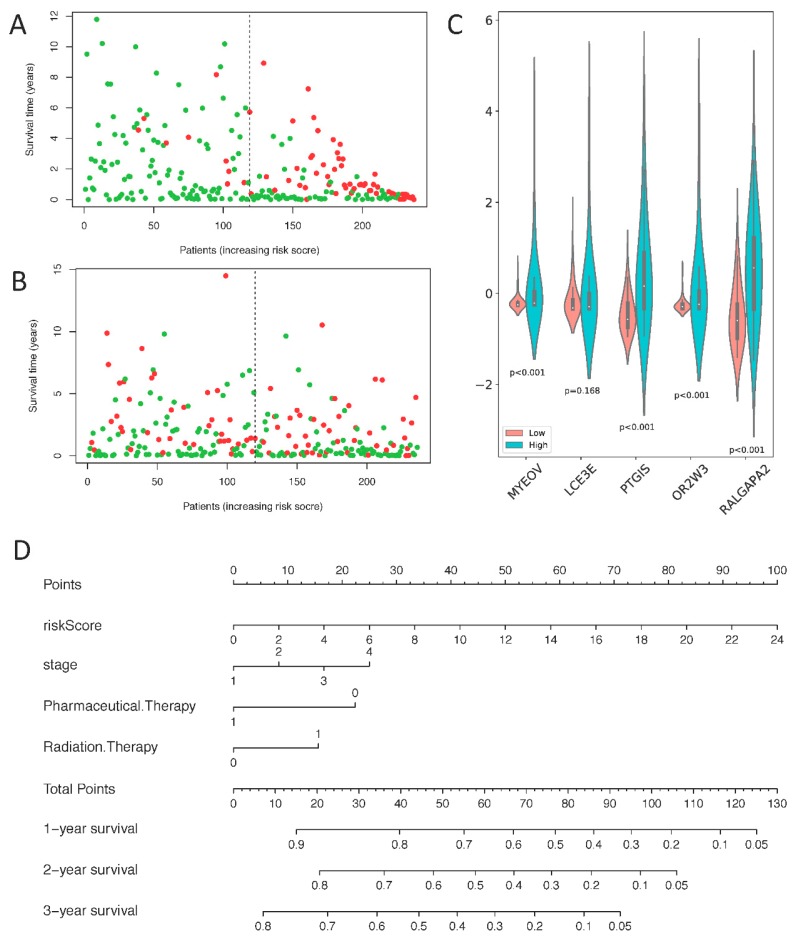
(**A**)Distribution of duration of survival in the training data. The x-axis is arranged in order of patient risk score and y-axis represents patient survival time. (**B**) Distribution of duration of survival in the test data. (**C**) The expression of 5 prognostic genes in the test data, blue is the high-risk group, and red is the low-risk group. (**D**) A nomogram for prognostic judgment. “Points” is a scoring scale for each factor, and “total points” is a scale for total score. Based on the total score of the patient, the 1-, 2- and 3-years survival rate can be inferred. In “Pharmaceutical.Therapy”, 1 represents treatment, 0 represents no treatment; “Radiation.Therapy” represents 1 treatment, and 0 represents no treatment.

Scatter plots were plotted based on survival time and risk scores. As risk scores increased, the number of patients that died increased and duration of survival gradually decreased in both training (Figure 6A) and test data (Figure 6B). The results above demonstrate that the definition of “risk score” is effective. In the training (Supplementary Figure S7F) and test data (Figure 6C), MYOEV, PTGIS, OR2W3, and RALGAPA2 genes were significantly highly-expressed in the high–risk group (*p* < 0.001), consistent with their positive coefficients in the risk score formula (1). The difference in LCE3E gene expression between the high–risk and the low–risk groups was not significant in either training or test data. A similar trend was also observed in the heatmap of gene expression (Supplementary Figure S7D–E).

### 2.6. Validation of Prognostic Model

At present, American Joint Committee on Cancer (AJCC) stage is often used to evaluate the prognostic effect of cancer. In addition, Carter et al. [21] identified a signature of chromosomal instability (CIN25) from specific genes (containing 25 genes) whose expression was consistently correlated with total functional aneuploidy. The method proved to be useful for prognosis assessment of various cancers [22,23]. We collected the AJCC stage of LUSC patients in the TCGA database, and then we calculated the CIN25 of the patients. In order to further verify the effectiveness of the risk score and compare it with AJCC stage and CIN25, we calculated the C index of the risk score, AJCC stage and CIN25 (Table 1). The C index of the risk score in the test set is 0.642, which is significantly greater than that of AJCC stage (0.576, *p* < 0.05) and CIN25 (0.555, *p* < 0.05); the C index of the risk score in the training set is 0.668, which is significantly greater than the C index of AJCC stage (0.527, *p* < 0.05) and CIN25 (0.545, *p* < 0.05). This illustrates that compared with other prognostic methods, the risk score is a good predictor of the patient’s prognostic status.

**Figure 7:**
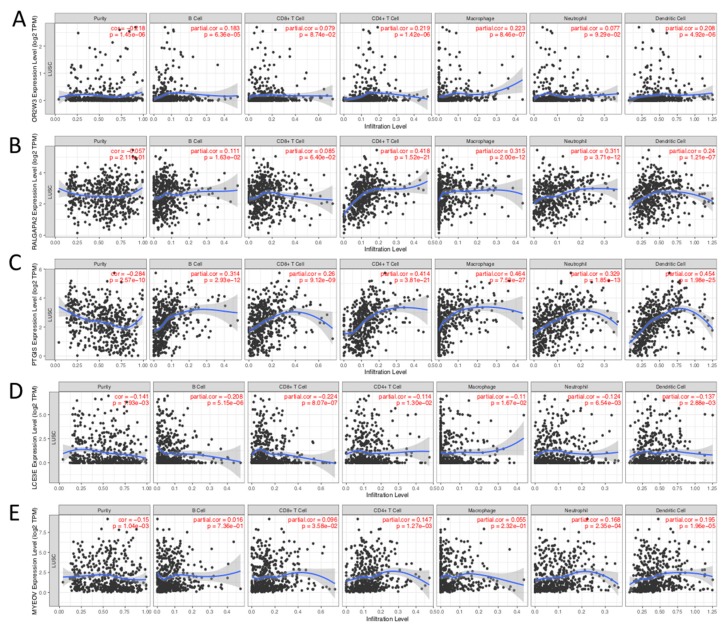
Correlation between different expressed prognostic genes and immune cell infiltration (TIMER). The correlation between the abundance of immune cell and the expression of (**A**) OR2W3, (**B**) RALGAPA2, (**C**) PTGIS, (**D**) LCE3E, (**E**) MYEOV in LUSC. “Purity” represents the purity of the tumor cells in the sample.

We further analyzed whether SCNA and the degree of cancer’s immune invasion is closely related to the prognosis. Four out of five genes don’t show any correlation between SCNA and gene expression (Supplementary Table S8). In order to further explore the function of genes in the risk score, we used the TIMER database (https://cistrome.shinyapps.io/timer/) to assess the correlation between prognostic gene expression and immune infiltration. There was a positive correlation between OR2W3 expression and the infiltration of B cells (Cor = 0.183, *p* = 6.36e-05), CD4+ T cells (Cor = 0.219, *p* = 1.42e-06), Macrophage cells (Cor = 0.223, *p* = 8.46e-07) and Dendritic cells (Cor = 0.208, *p* = 4.92e-06; Figure 7A). RALGAPA2 expression was positively associated with the infiltration of B cells (Cor = 0.111, *p* = 1.63e-02), CD4+ T cells (Cor = 0.418, *p* = 1.52e-21), macrophage cells (Cor = 0.315, *p* = 2.00e-12), neutrophil cells (Cor = 0.311, *p* = 3.71e-12) and dendritic cells (Cor = 0.240, *p* = 1.21e-07; Figure 7B). PTGIS expression was positively associated with the infiltration of B cells (Cor = 0.314, *p* = 2.93e-12), CD8+ T cells (Cor = 0.260, *p* = 9.12e-09), CD4+ T cells (Cor = 0.414, *p* = 3.81e-21), macrophage cells (Cor = 0.464, *p* = 7.53e-27), neutrophil cells (Cor = 0.329, *p* = 1.85e-13) and dendritic cells (Cor = 0.454, *p* = 1.98e-25; Figure 7C). LCE3E expression was negatively associated with the infiltration of B cells (Cor = -0.208, *p* = 5.15e-06), CD8+ T cells (Cor = -0.224, *p* = 8.07e-07), CD4+ T cells (Cor = -0.114, *p* = 1.30e-02), macrophage cells (Cor = -0.11, *p* = 1.67e-02), neutrophil cells (Cor = -0.124, *p* = 6.54e-03) and dendritic cells (Cor = -0.137, *p* = 2.88e-03; Figure 7D). MYEOV expression was positively associated with the infiltration of CD4+ T cells (Cor = 0.147, *p* = 1.27e-03), neutrophil cells (Cor = 0.168, *p* = 2.35e-04) and dendritic cells (Cor = 0.195, *p* = 1.96e-05; Figure 7E). The above results indicate that the 5 genes in the risk score formula are closely related to the immune infiltration process of LUSC patients, which may be one reason they can be used as effective prognostic markers.

## 3. Discussion

Although many relevant studies of LUSC have been performed, early diagnosis, efficacy of treatment and prognosis for LUSC remain poorly resolved. For diagnosis and treatment, it is necessary to further understand the molecular mechanisms resulting in occurrence and development. Due to the development of high–throughput sequencing technology, the genetic alterations due to disease progression can be detected, indicating gene targets for diagnosis, therapy and prognosis of specific diseases.

In this study, by intergrading of GEO and TCGA data for LUSC and combining the PPI and WGCNA analysis method, we finally identified four genes as hub genes for the occurrence and development of LUSC. Remarkably, the WGCNA constructed a network based on the correlation between genes, whereas the PPI network was based on protein networks reported in the known literature. It seems appropriate to combine WGCNA and PPI methods to identify key genes. All four genes are involved in cell cycle progression and regulation of different mitotic events. A large number of studies have reported that abnormal expression levels were found in multiple types of human malignancy and have potential as anticancer therapeutic targets.

Cyclin family member CCNA2 is involved in cytoskeletal dynamics, epithelial–mesenchymal transition (EMT) and metastasis [24]. Chen et al. used two lung adenocarcinoma cell lines to verify that miR-137 can induce G1/S cell cycle arrest and dysregulate mRNA expression in cell cycle associated proteins, including CCNA2 [25]. AURKA and AURKB are members of the aurora kinase family and both play central roles in regulating cell-cycle progression from G2 to cytokinesis. AURKA is involved in many mitotic events, including centrosome maturation, mitosis entry, mitotic spindle formation and cytokinesis [26]. AURKB exerts its function by regulating chromosomal alignment, segregation and cytokinesis, as the catalytic protein of the chromosomal passenger complex (CPC) [27]. Yu et al. identified functional interaction between AURKA and AURKB, assisting in protection of their stability and partially explaining their persistent high expression and activity in cancers [28]. Ma et al. verified that up–regulation of miR-32 down–regulated AURKA and thus inhibited the occurrence and development of NSCLC [29]. Jin et al. performed a bioinformatics analysis and proposed that AURKB may be the key gene in LUAC and could result in poor prognosis [30]. FEN1 belongs to the Rad2 structure-specific nuclease family and plays a crucial role in maintaining genome stability, DNA replication and repair. Over expression of FEN1 has been found in many cancers and it can influence the cell proliferation and differentiation [31]. Meanwhile, some reports also pointed that FEN1 expression influence on tumor cell growth to anticancer drugs, it can accelerate tumor cell growth and confers cisplatin resistance in NSCLC [32]. The correlation between SCNA and high gene expression have not found in these four hub genes, although the SCNA of AURKA, AURKB, and CCNA2 in LUSC does exist. The correlation between high gene expression and biological factors need further analysis.

We have established a prognostic model to predict patient survival rate. This model contains 5 key genes, namely OR2W3, RALGAPA2, PTGIS, LCE3E, and MYEOV. OR2W3 is an important member of the olfactory receptor (OR) family, mainly expressed on the surface of neurons in the olfactory epithelium [33]. However, previous studies have found that OR family proteins are located in prostate and renal tubular epithelial cells [34,35], indicating that its function is not limited to smell. OR2W3 is a cell division promoting factor, which shortens the time to progress from G0 to G1 and increases the degree of cell proliferation [36]. The expression of OR2W3 can promote dysplasia and local aggressive growth of tumor cells. A number of researchers have observed high expression of OR2W3 in pancreatic tumors, suggesting that OR2W3 may play an important role in regulating pancreatic epithelial dysplasia and participate in complex changes of oncogenes and tumor suppressor genes [37]. Although OR2W3 has not so far been reported in LUSC at present, indicating the OR2W3 gene has the largest coefficient (0.180) in the risk score formula, which shows that OR2W3 is a very important prognostic factor in LUSC, representing an important reference for judging the prognosis of patients.

Ral GTPase activating protein catalytic subunit alpha 2 (RALGAPA2) is highly expressed in lung tissue [38]. Ral is a member of the Ras-like GTP hydrolase family [39]. The RAS family of proteins is a class of small-molecule GTPases and the first oncogene found in human tumor cells and widely involved in cell growth, differentiation and tumorigenesis and tumor development [40]. GTPase activating proteins (GAPs) and a class of proteins called guanine nucleotide exchange factors (GEFs) can jointly regulate the Ral pathway. GAPs generally function as tumor suppressor genes and can promote hydrolysis of Ral GTPase activating protein to inhibit tumorigenesis and development [41]. RALGAPA2 is located upstream of the tumor regulatory pathway and playing a key role, although it has not been reported in LUSC, and further research is required to determine its importance in this cancer.

Prostaglandin I2 synthase (PTGIS) is a rate–limiting enzyme for Epoprostenol (PGI2) [42]. PGI2 promotes relaxation of vascular smooth muscle, inhibits cell proliferation and platelet aggregation by binding specifically to the prostacyclin receptor and then activating the downstream pathway [43]. Related studies [44] have shown that the expression levels of PTGIS and PGI2 in lung cancer are significantly higher than those in the normal control group, which is consistent with our results. The expression levels in the high-risk group were significantly higher than those in the low-risk group.

Myeloma overexpressed gene (MYEOV) is located on chromosome 11q13, this region has been reported to have frequent DNA amplification. Up-regulated MYEOV is associated with poor prognosis has been verified in many carcinomas, including esophageal squamous cell carcinoma, breast cancer in addition to NSCLC. In NSCLC, MYEOV exerts an influence on invasion and metastasis, the principal cause for poor prognosis [45,46]. Late cornified envelope 3E (LCE3E) is mainly involved in the keratinization pathway and the formation of the cornified envelope [47]. The overexpression of this gene may have strong connection with squamous cell carcinoma.

We found that as risk score improves, “points” can often have a great improvement as shown in nomogram (Figure 6D), indicating that compared with the clinical information, risk score has a greater impact on prognosis. Medication (“Pharmaceutical.Therapy” = 1) can effectively improve survival rate of patients. It is more interesting that radiotherapy (“Radiation.Therapy” = 1) reduces survival in patients, which may be because radiotherapy suppresses the function of the lung. Side effects of radiotherapy include fatigue and anxiety. Furthermore, radiotherapy also often reduces the number of white blood cells and platelets, and suppresses immune function, causing patients to be more vulnerable to infection. Therefore, radiotherapy in patients with LUSC should be performed with caution.

## 4. Materials and Methods

### 4.1. Data Collection and Data Processing

The Gene Expression Omnibus (GEO, https://www.ncbi.nlm.nih.gov/geo/) database from the National Center for Biotechnology Information was searched for publicly available studies and samples that fulfilled the following criteria for analysis: (1) The gene expression data series contained LUSC tissue and normal tissue samples; (2) the species of the samples was *Homo sapiens*; (3) the information of each sample should be consistent and without missing values. Finally, three gene expression profiles (GSE2088, GSE6044, GSE19188) were collected for further analysis [48]. To eliminate the batch effect of three datasets, R package “sva” were applied. If multiple probes corresponded to the same gene, the maximum value of expression was considered the gene expression level [49].

To increase the robustness, LUSC RNAseq data was downloaded from The Cancer Genome Atlas (TCGA, https://www.cancer.gov/tcga) database, acquired using the Illumina Hiseq platform. The dataset included 502 LUSC samples and 49 normal samples. The genomic data used was standardized using the fpkm method [50]. For repetitive genetic data, the largest value was utilized as the expression level. Genes with a mean expression of less than 0.5 were excluded in each case to ensure significantly expressed genes were evaluated.

In both datasets, the *p*-values of differentially–expressed genes were identified using a Wilcox test. Raw *p*-values obtained from the Wilcox test and log2FC calculation were adjusted using the BH16 method [51]. Thresholds of |log2FC| > 1.0 and an adjusted *p*-value of < 0.05 were selected. The screening process was conducted using the R package “limma”.

### 4.2. Functional and Pathway Enrichment Analysis

The GO database has a large collection of gene annotation terms, allowing genome annotation using consistent terminology. GO enrichment analysis including molecular function (MF), cellular components (CC) and biological processes (BP), identified which GO terms were over or underrepresented within a given set of genes [52]. The KEGG knowledge database, an integrated database resource, is generally used to identify functional and metabolic pathways [53]. GO and KEGG analysis were both conducted using the Database for Annotation, Visualization and Integrated Discovery (DAVID, Frederick, USA. https://david.ncifcrf.gov/), analysis tools for extracting meaningful biological information from multiple gene and protein collections [54,55]. Up- and down-regulated genes were analyzed separately, a *p*-value < 0.05 considered the threshold value.

### 4.3. PPI Network Construction and Analysis of Modules

Protein–protein interaction (PPI) networks can assist in identifying key genes and pivotal gene modules involved in the development of LUSC from an interaction level. The Search Tool for The Retrieval of Interaction Genes (STRING, Zurich, Switzerland, https://string-db.org/) was used to construct PPI networks. STRING is a database which provides critical assessment and integration of protein–protein interactions, including physical and functional associations [56]. DEGs were mapped to STRING to evaluate the PPI information and set confidence score > 0.4 as the cut-off standard. Cytoscape was used to visualize the PPI network, a practical open–source software tool for the visual exploration of biomolecule interaction networks consisting of protein, gene and other types of interaction [23]. Five methods in Plug-in CytoHubba were used to select the key genes in PPI, namely EPC (Edge percolated component), MCC (Maximal clique centrality), MNC (Maximal neighborhood component), Degree (Node connect degree) and Closeness (Node connect closeness). Top 20 genes in each method were selected and then intersection was taken to get the key genes in PPI analysis. Module analysis was conducted using the plug-in Molecular Complex Detection (MCODE) in Cytoscape to display the biological significance of gene modules [57].

### 4.4. Weighted Correlation Network Analysis of DEGs

In the WGCNA algorithm [58], the power exponential weighting of gene correlation coefficients was used to represent the correlation between genes. The power value *β* was selected such that connections between genes were subject to scale-free network distribution. A topological matrix that incorporated surrounding genetic information was calculated relative to distance *d*.

$$S_{ij}=\mathrm{cor}(i,j)\;\alpha_{ij}={\left|S_{ij}\right|}^{\beta }\;\omega_{ij}=\frac{l_{ij}+a_{ij}}{\min\left\{k_{i},\;k_{j}\right\}+1-a_{ij}}\;d_{ij}=1-\omega_{ij}$$

where *l_ij_* = ∑*_u_ α_iu_α_uj_* and *k_i_* = ∑*_u_ α_iu_* represented node connectivity. The samples were firstly clustered using hierarchical clustering and the threshold set to 40,000 to eliminate outliers. *d* was then calculated and the dynamic pruning method was used after determining values of gene module parameters (maxBlocksize = 7000, deepSplit = 2, minModuleSize = 80, mergeCutHeight = 0.25). After obtaining these, Pearson correlation coefficients of the gene modules and phenotypes (cancer tissue or paracancerous tissue samples) were calculated. This allowed selection of gene modules closely related to tumorigenesis. Genes in the gene modules from GO and KEGG analysis were used to observe the function of each gene module. Next, two indicators for the genes in these gene modules were calculated: one was the Pearson correlation coefficient of the gene in the module and the first principal component of the module, termed Module Membership (MM); the other is the Pearson correlation coefficient of the gene and phenotype in the sample, termed Gene Significance (GS). If a gene within a module had both large MM and GS, then the gene was considered to be a key gene in the module. The WGCNA calculations were accomplished using the “WGCNA” package in R [59].

### 4.5. Finding Hub Gene and Verification

In this study, genes in which the MM and GS were in the upper quartile of all genes in the module were identified as key genes. The key genes in key modules (modules with a high correlation coefficient for phenotype) were compared with key genes derived from PPI to obtain decisive genes in the development of LUSC. For further validation of our analysis, immunohistochemistry (IHC) datasets (The Human Protein Atlas database) was used to verify.

### 4.6. Survival Analysis

Patient clinical data were downloaded from the TCGA website, then samples missing overall survival (OS) data were deleted, finally resulting in a total of 473 samples for survival analysis. In order to make the established prognostic model have better generalization ability, we randomly divide the samples into training group (237 samples) and test group (236 samples). We used the training dataset to build the model and verified it in the test group. Univariate Cox proportional hazards regression analysis was used to screen for genes significantly associated with prognosis (*p*< 0.05) [60]. Later, lasso regression analysis was used to eliminate collinearity between genes. After performing 1000 10-fold cross-validations, the *λ* value in which the error was minimized was selected as the optimum *λ* parameter value [61]. Then, multivariate Cox proportional hazards regression analysis was used to find key genes involved in the establishment of a prognostic model [62]. The model used disease risk scores as predictors of prognostic status. The disease risk score was determined by parameter *β* of the multivariate Cox proportional hazards regression analysis and the magnitude of the expression of each gene in the sample, using the following formula:

$$\mathrm{Risk}\;\mathrm{score}=\overset{N}{\underset{i=1}{\sum }}Expi\times \beta_{i}$$

where *Expi* and *β* are the expression levels of gene *i* in a particular patient and coefficients of the gene *i* in the multivariate cox regression analysis [63]. The risk score for each patient was calculated, which were then categorized into high or low–risk in comparison with the median value. Kaplan–Meier survival curves were then plotted to evaluate whether the prediction effect of the model was significant (*p* < 0.05). The statistical method used in this process was a Log-rank test. The predictive performance of this model at different endpoints (1, 3, or 5 years) was assessed using a time-dependent receiver operating characteristic (ROC) curve [64]. The purpose of the above verification methods is to show that the risk score we get is reasonable. In addition, in order for our model to be more effectively applied to the clinical process, we took the clinical information (synchronous malignancy, gender, age, stage, chemotherapy, radiotherapy) into the prognostic model. Removing the samples with missing clinical data, we obtained a total of 370 samples. We performed a multivariate independent prognostic analysis of risk score and clinical information. The key factors with a *p*-value of < 0.1 were found through the forward–backward selection method, and a nomogram was drawn. The R packages used in the survival analysis procedure included: “survival”, “caret”, “glmnet”, “survminer", and “survivalROC”.

## 5. Conclusions

In the present study, microarray data from the GEO database were integrated with RNA sequencing data from the TCGA, to identify key genes and more important hub genes. Finally, we identified four hub genes associated with the pathogenesis and progression of LUSC. These genes all play a crucial role in cell cycle and its abnormal behavior in lung cancer as reported, which indicate that these genes have huge potential as targets in LUSC treatment. Additionally, we performed survival analysis and built a Cox proportional hazards model to identify prognostic biomarkers. A prognostic gene signature consisting of 5 genes was constructed with good performance in predicting overall survival. These results will serve as a reference for future research on the pathogenesis and drug treatment for LUSC. Nevertheless, although the authentication has been done by using the other database and clinical data, the lack of experimental verification is still a limitation of this study. The predictions obtained from the bioinformatics analysis can be verified by future experimental studies.

## Figures and Tables

**Table 1:** C index of risk score, American Joint Committee on Cancer (AJCC) stage and chromosomal instability (CIN25).

Method	Training Set		Test Set	
	C–index(95%CI)	*p*	C–index(95%CI)	*p*
Risk score	0.668(0.611,0.725)	–	0.642(0.580,0.706)	–
AJCC stage	0.527(0.448,0.605)	<0.05	0.576(0.496,0.656)	< 0.05
CIN25	0.545(0.469,0.621)	<0.05	0.555(0.486,0.624)	< 0.05

## Supplementary Materials

The following are available online at https://www.mdpi.com/1422-0067/21/8/2994/s1.

## Abbreviations

SCLC	Small Cell Lung Cancer
NSCLC	Non–Small Cell Lung Cancer
LUAD	Lung Adenocarcinoma
LUSC	Lung Squamous Cell Carcinoma
DEGs	Differentially Expressed Genes
PPI	Protein–protein interaction
FC	Fold Change
GEO	Gene Expression Omnibus
TCGA	The Cancer Genome Atlas
GO	Gene Ontology
KEGG	Kyoto Encyclopedia of Genes and Genomes
DAVID	Database for Annotation, Visualization and Integrated Discovery
STRING	Search Tool for The Retrieval of Interaction Genes
MCODE	Molecular Complex Detection
WGCNA	Weighted Gene Co–Expression Network Analysis
GS	Gene Significance
MM	Module Membership
OS	Overall Survival
Gene Abbreviation	
CCNA2	Cyclin A2
AURKA	Aurora Kinase A
AURKB	Aurora Kinase B
FEN1	Flap endonuclease–1
OR2W3	Olfactory Receptor Family 2 Subfamily W Member 3
RALGAPA2	Ral GTPase activating protein catalytic subunit alpha 2
PTGIS	Prostaglandin I2 synthase
MYEOV	Myeloma overexpressed gene
LCE3E	Late cornified envelope 3E
