# Supplementary material for: Identification of Key Genes Related to Lung Squamous Cell Carcinoma Using Bioinformatics Analysis
Source: Int J Mol Sci. 2020 Apr 23;21(8):2994. doi: 10.3390/ijms21082994 (PMC7215920; doi:10.3390/ijms21082994)
Supplement: Supplementary file 1 [file ijms-21-02994-s001.pdf]

Supplementary data

Supplementary Table S1. Detailed information of the GEO datasets.

GEO Accession	Platform	Number of LUSC sample	Number of normal sample	Total
GSE2088	GPL962	48	28	76
GSE6044	GPL201	9	4	13
GSE19188	GPL570	27	65	92
In all	-	84	97	181

Supplementary Table S2. Detailed information about PPI cluster by MCODE analysis.

Cluster Number	MCODE Score	Nodes	Edges
1	52.075	54	1380
2	7.6	61	228
3	7.143	22	75
4	5	5	10
5	4.48	26	56
6	4.222	10	19

Supplementary Table S3. MM and GS cutoff of three significant modules.

cut-off	yellow	blue	turquoise
**MM**	0.756	0.683	0.708
**GS**	0.431	0.39	0.631

Supplementary Table S4. All scores by different methods of significant genes.

Gene	EPC	MCC	MNC	Degree	Closeness	MM	GS
CCNA2	166.175	5.37E+46	85	85	247.066667	0.69935938	0.44450364
AURKA	165.8	5.37E+46	78	78	231.833333	0.73247711	0.46916748
AURKB	165.585	5.37E+46	77	78	229.283333	0.69979796	0.4295527
FEN1	165.558	5.37E+46	78	78	228.716667	0.75210615	0.43750092

Supplementary Table S5. Detailed information about IHC results of four hub genes.

Gene	Cancer Grade	Gender	Age	Antibody	Antibody Staining	Intensity	Quantity
**CCNA2**	High	Male	64	CAB000114	Medium	Moderate	75%-25%
	Low	Male	65	CAB000114	Not detected	Negative	None
**AURKA**	High	Male	71	HPA002636	Low	Moderate	<25%
	Low	Male	69	HPA002636	Not detected	Negative	None
**AURKB**	High	Male	71	CAB005862	High	Strong	75%-25%
	Low	Male	69	CAB005862	Not detected	Negative	None
**FEN1**	High	Male	68	CAB002262	High	Strong	>75%
	Low	Male	69	CAB002262	Low	Moderate	<25%

Supplementary Table S6. P value represents the degree of the correlation between hub genes' expression and SCNA. FEN1 had not found SCNA in LUSC. P value < 0.05 means there is correlation.

Gene Name	P value
CCNA2	0.47895054
AURKA	0.49158095
AURKB	0.49158095
FEN1	-

Supplementary Table S7. Multi-factor independent prognostic analysis results.

ID	HR	HR.95L	HR.95H	P value
**risk**	2.31387722	1.55124049	3.45144922	3.92E-05
**age**	1.01188332	0.98556086	1.03890879	0.37970604
**gender**	0.98605616	0.62497076	1.55576357	0.951874
**stage**	1.30643285	1.01972308	1.67375519	0.0344757
**synchronous_malignancy**	1.59388583	0.21798358	11.6544194	0.64605341
**Pharmaceutical.Therapy**	0.64114346	0.38091166	1.07916079	0.09428921
**Radiation.Therapy**	1.57823702	0.94621507	2.63241641	0.08043625

Supplementary Table S8. P value represents the degree of the correlation between prognosis gene expression and SCNA. OR2W3 had not found SCNA in LUSC. P value < 0.05 means there is correlation.

Gene Name	P value
OR2W3	-
RALGAPA2	0.49158095
PTGIS	0.49158095
MYEOV	0.49158095
LCE3E	9.75E-06

Supplementary Table S9. The parameters set in different classifiers used for the selection of best classification.

Classifier	Hyperparameter	Parameter set
RF	criterion	gini, entropy
	min_impurity_decrease	0.0001, 0.0005, 0.001, 0.002, 0.003,0.004, 0.005, 0.01, 0.02, 0.03, 0.04, 0.05
	min_samples_leaf	2, 3, 5, 6, 7, 8, 9, 10, 11
RF	max_features	auto, sqrt, log2
	n_estimators	10, 50, 100, 200, 300, 400, 500, 600, 700, 800, 900

Supplementary Table S10. The best parameters for each model.

Classifier	Hyperparameter	1st year	3rd year	5th year
DT	criterion	gini	gini	gini
	max_depth	2	4	4
	min_impurity_decrease	0.0001	0.001	0.005
	min_samples_leaf	5	5	2
RF	max_features	auto	auto	auto
	n_estimators	600	100	50

Supplementary Figure

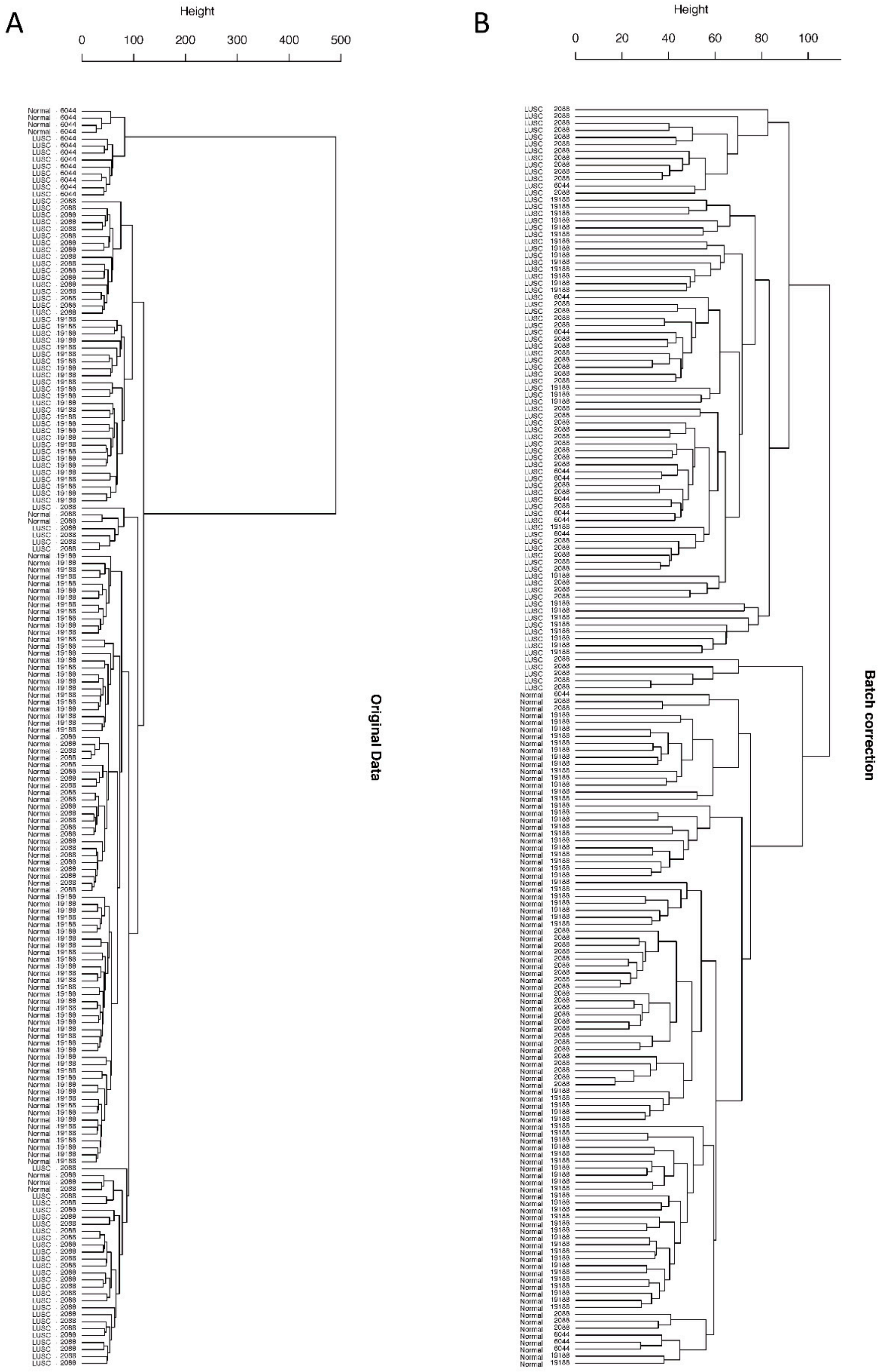

Supplementary Figure S1. Hierarchical clustering of three microarray samples in GEO database. (A) Original expression samples. (B) Samples after batch correction.

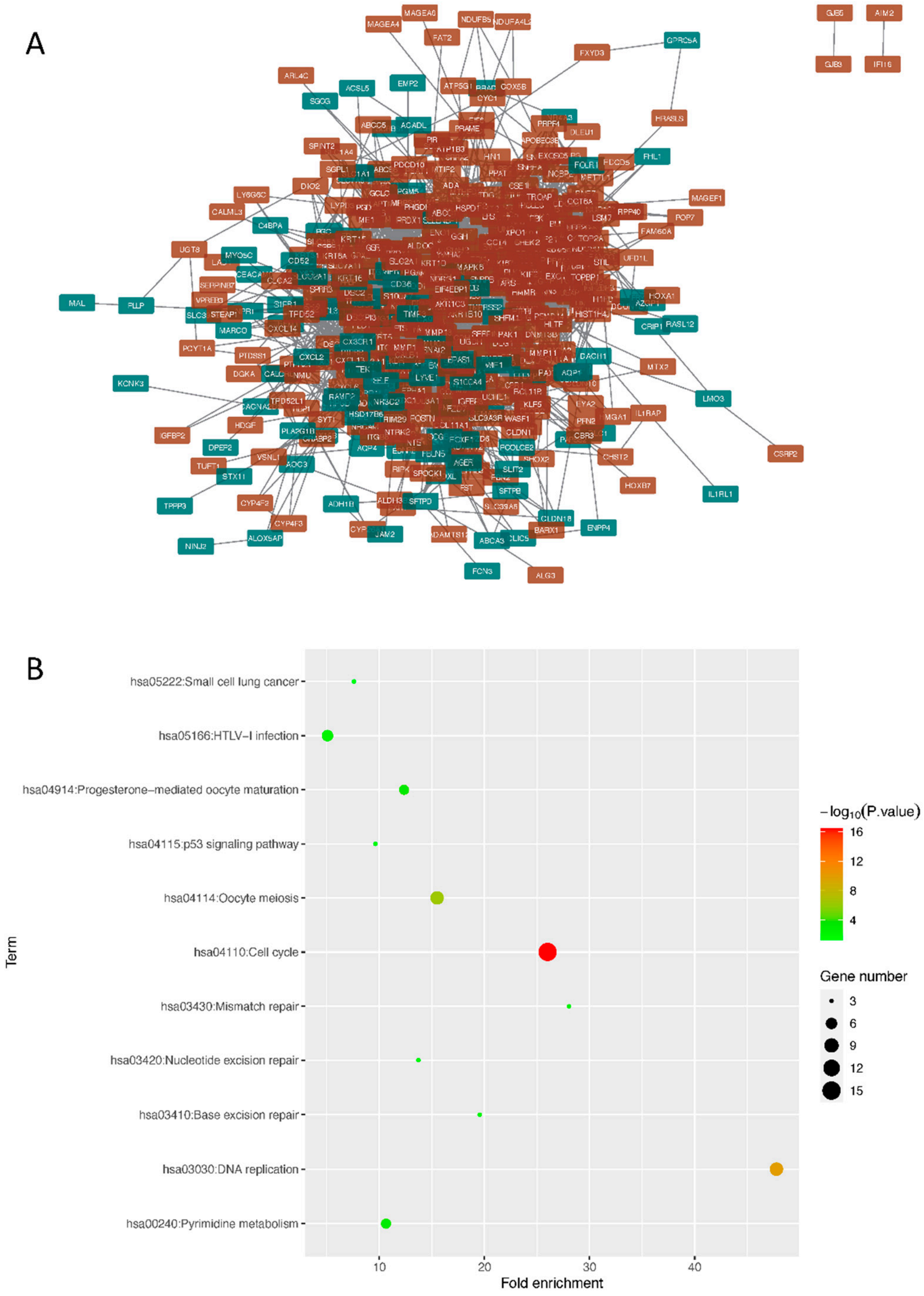

Supplementary Figure S2. (A) PPI network of 476 significantly differentially expressed genes. Red: up-regulated genes; Green: down-regulated genes; (B) KEGG analysis of the most significant module in PPI analysis. The bubble size represents the number of genes, and the bubble color represents the magnitude of the significance. The abscissa is the degree of enrichment, and the ordinate is the different regulatory pathway items.

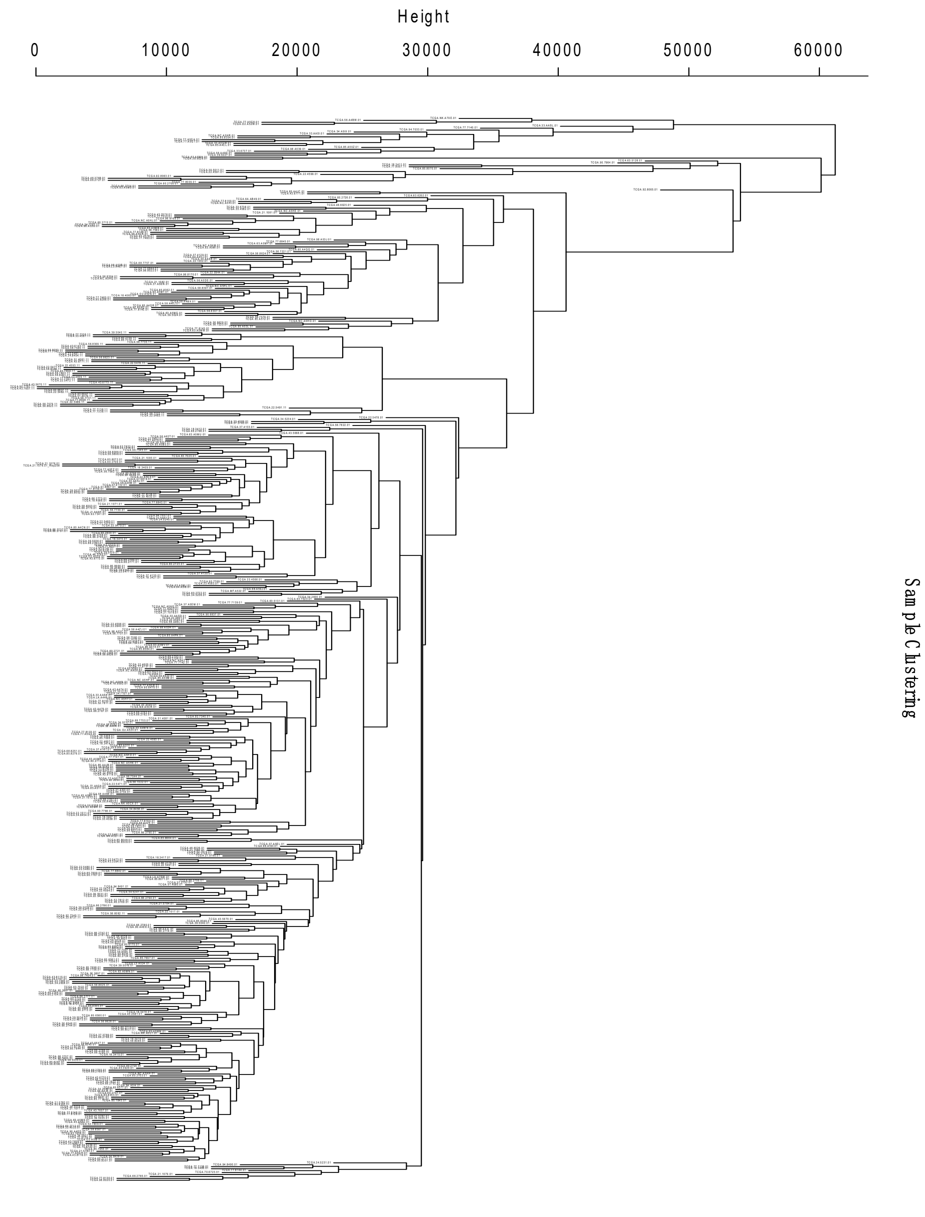

Supplementary Figure S3. A tree diagram of the hierarchical clustering results before the sample is removed.

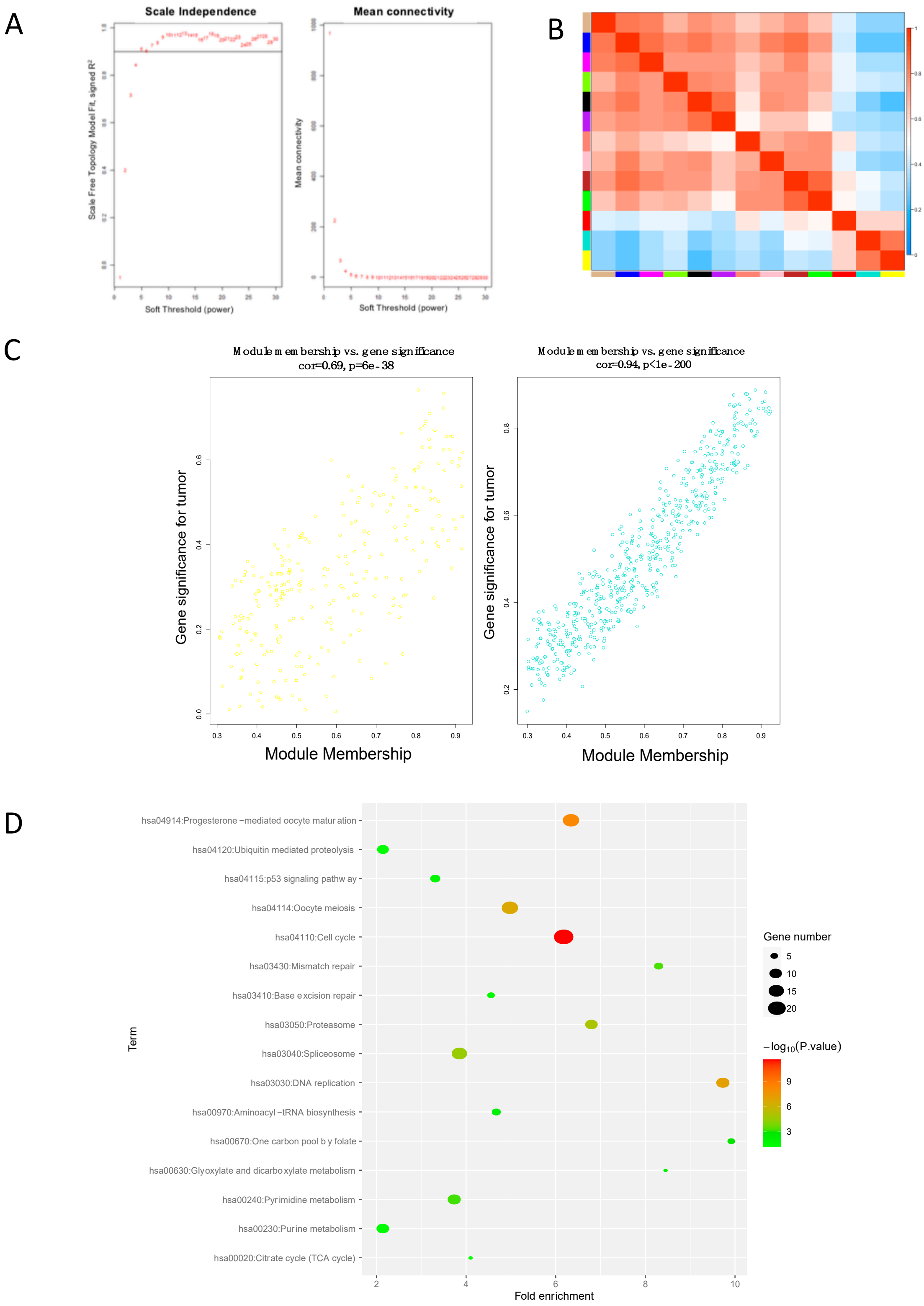

Supplementary Figure S4. (A) Soft-cutoff screening. Left panel: Abscissa: power value; ordinate: R2 value after linear regression of -log10 (k) and log10 (P (k)). k: connectivity of the gene nodes; P (k): probability of such a node. Horizontal line: 0.9. Right panel: Abscissa: power value; ordinate: mean connectivity of gene nodes. (B) Interrelationship between different gene modules. Colors in the heat map represent Pearson correlation coefficients between gene

modules. Expression levels of gene modules are represented by the first principal component. (C). Yellow and turquoise module gene correlation scatter plots. X-axis represents molecule membership, i.e. Pearson correlation coefficients of gene and module (MM). Y-axis represents the importance of the gene for the phenotype, i.e. Pearson's correlation coefficient of gene and phenotype (GS: phenotype is represented by a Boolean variable). In the yellow, turquoise modules, the upper quartile value of MM was 0.756 and 0.708 respectively; the upper quartile value of GS was 0.431 and 0.631, respectively. (D) KEGG analysis of the blue module. The bubble size represents the number of genes, and the bubble color represents the magnitude of the significance. The abscissa is the degree of enrichment, and the ordinate is the different regulatory pathway items.

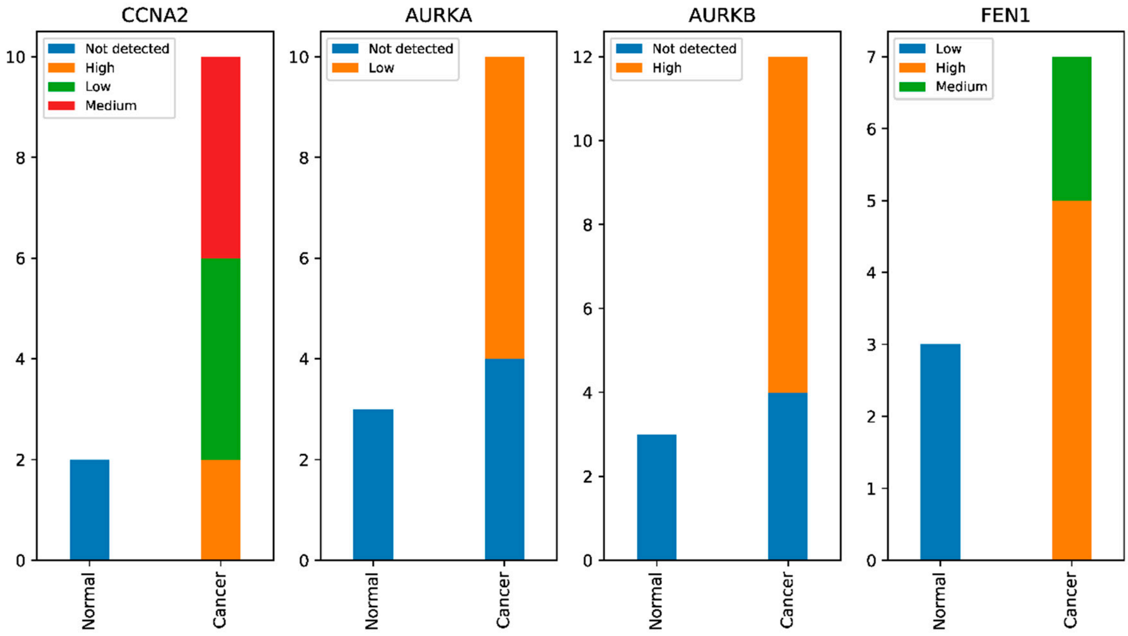

Supplementary Figure S5. The four genes' antibody staining distribution of LUSC and normal tissue in the Human Protein Database. Y-axis represents the number of samples.

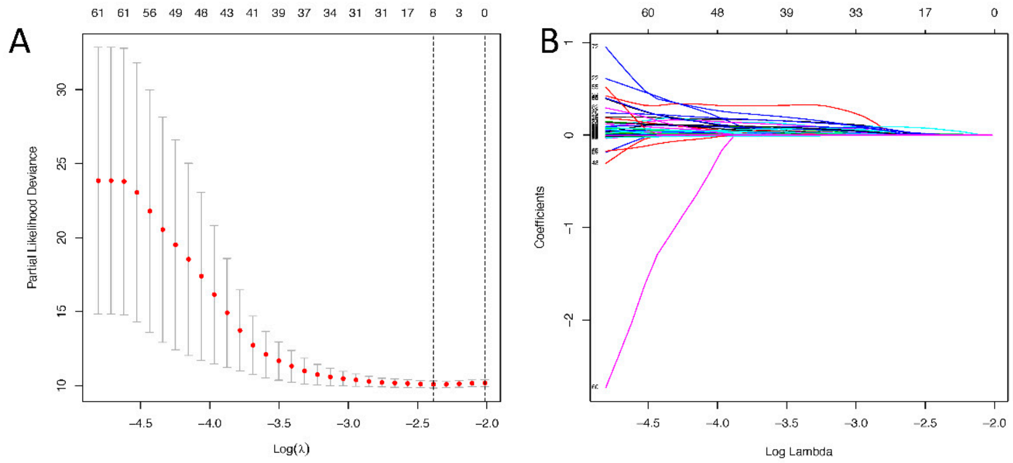

Supplementary Figure S6. (A)Results of Lasso regression 1000 times 10-fold cross validation. λ was determined when partial likelihood deviance was smallest. (B) Coefficient curve. Different colored lines represent coefficient sizes of individual genes in different cases. The abscissa represents log (λ) and the number of coefficients (top) that are not zero under this penalty factor.

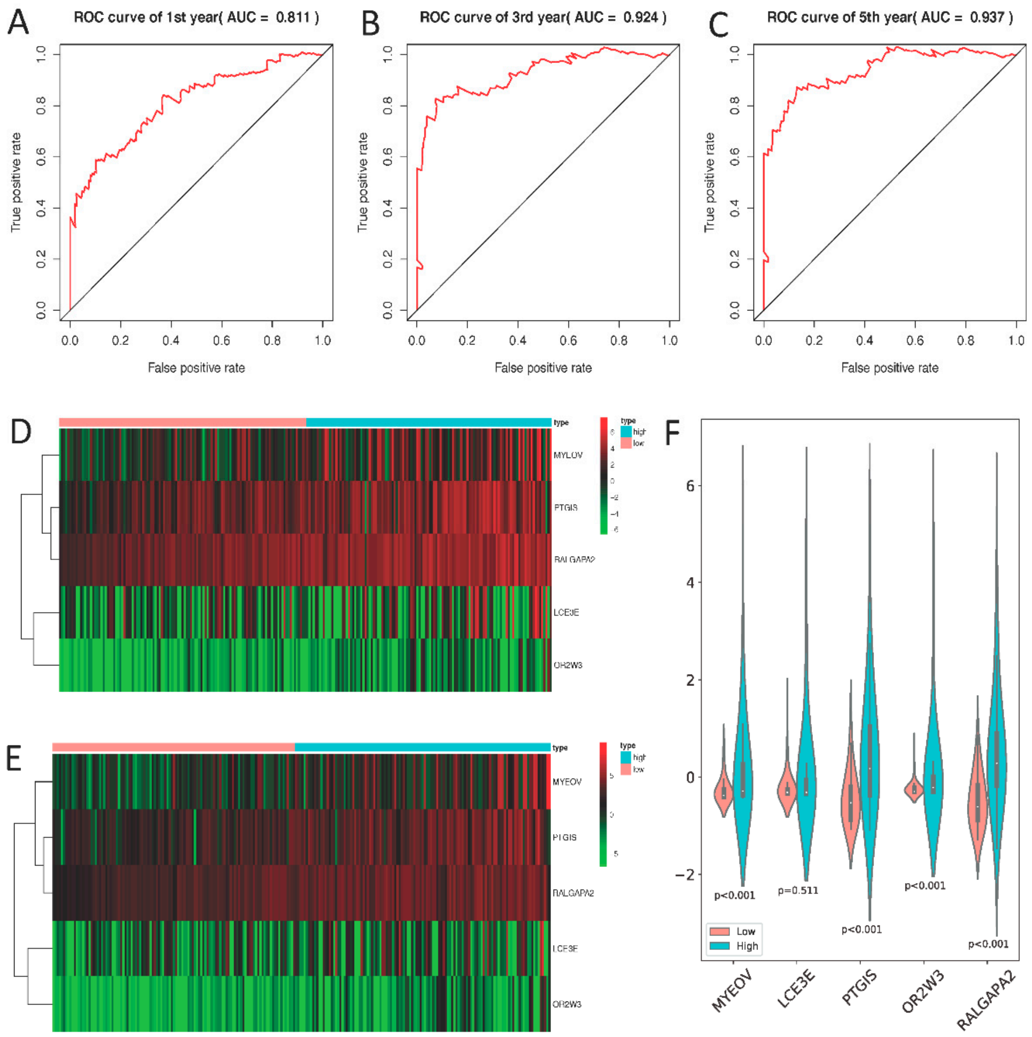

Supplementary Figure S7. (A-C) ROC curves for the model representing 1, 3 and 5-year predictions in training data, respectively. The values in brackets are the areas under the curve. (D) Heat map of gene expression levels of 5 prognostic genes in the training data. Patient risk scores increased from left to right. (E) Heat map of gene expression levels of 5 prognostic genes in the test data. (F) The expression of 5 prognostic genes in the training data, blue is the high-risk group, and red is the low-risk group.

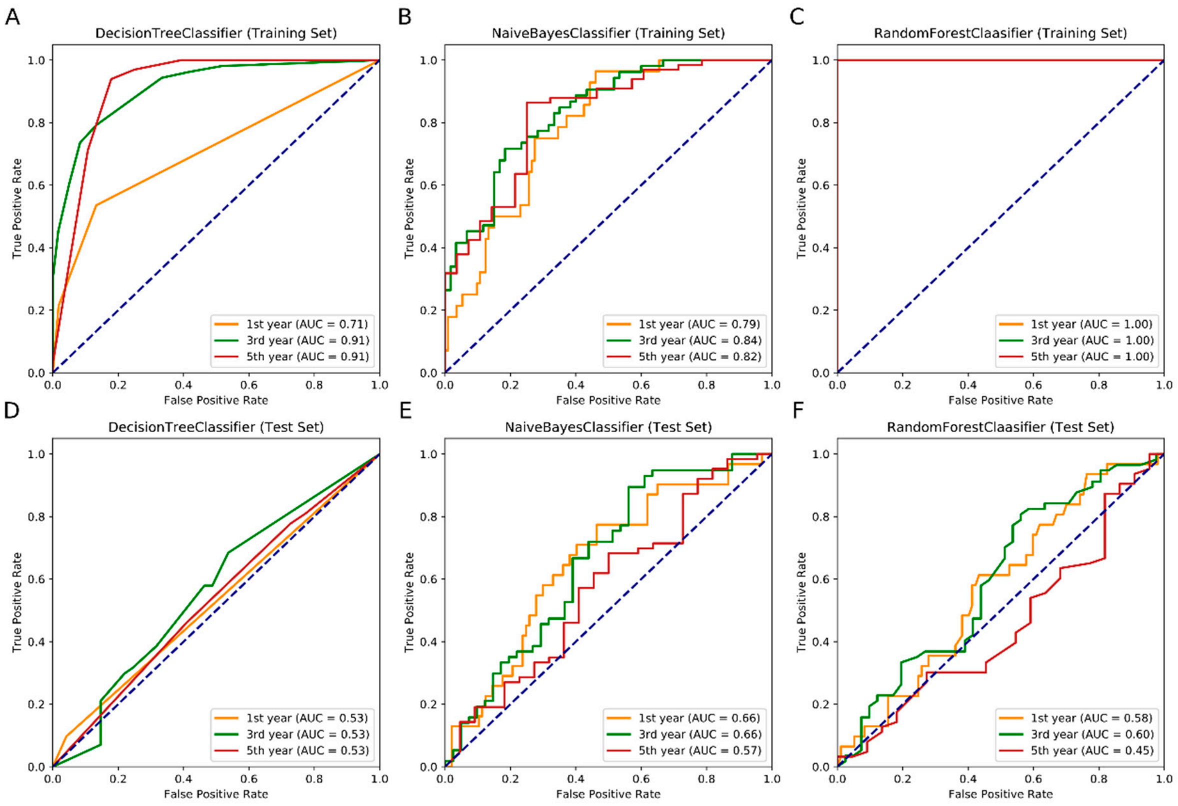

Supplementary Figure S8. The receiver operating characteristic curve (ROC) of machine learning algorithms to predict 1-, 3-, 5-years survival rate in training set and test set.

Supplementary method

Expect for linear regression, we now tested other several machine learning algorithms, including Decision Tree (DT), Naïve Bayes (NB), and Random Forest (RF). We had used these methods and linear regression to predict 1–, 3–, 5–years survival rate and compare them. During this process, Python package GridSearch was also employed to select the best parameters for DT and RF classification (NB classification requires no hyperparameter). The detailed information of parameter sets was now shown in Supplementary Table S9. The best parameters for each classifier model in different data sets can be found in Supplementary Table S10.

All models showed an amazing AUC value on prediction of 1–, 3–, 5–years survival rate (Supplementary Figure S8 A–C) in training set. For DT classification, the AUC value for 1–, 3–, 5–years in training set are 0.71, 0.91, 0.91 respectively; For NB classification, that are 0.79, 0.84, 0.82; For RF classification, that is 1, which indicate the overfitting of this model. For the Cox regression model: that are 0.811, 0.924 and 0.937. However, the performance in test set looks not good (Supplementary Figure S8 D–F). For DT classification, the AUC value for 1–, 3–, 5–years are 0.53; For NB classification, the AUC value for 1–, 3–, 5–years are 0.66, 0.66, 0.57 respectively; For RF classification, the AUC value for 1–, 3–, 5–years are 0.58, 0.60, 0.45 respectively. The Cox regression model achieve a better performance than these models according to the comparation of AUC (0.692, 0.722, and 0.651).
